# Acute Megakaryoblastic Leukemia With Myeloid Sarcoma in the Temporal Bone: A Case Report

**DOI:** 10.7759/cureus.106041

**Published:** 2026-03-28

**Authors:** Nozomi Fujisawa, Takefumi Kamakura, Maki Yamashita, Kai Yamasaki, Yasuo Mishiro

**Affiliations:** 1 Otolaryngology, Osaka City General Hospital, Osaka, JPN; 2 Otorhinolaryngology, Osaka City General Hospital, Osaka, JPN; 3 Pediatrics, Adachi Hospital, Kyoto, JPN; 4 Pediatric Hematology and Oncology, Osaka City General Hospital, Osaka, JPN

**Keywords:** acute megakaryoblastic leukemia, computed tomography, down syndrome, myeloid sarcoma, temporal bone

## Abstract

We report a case of acute megakaryoblastic leukemia (AMKL) with myeloid sarcoma of the temporal bone. The patient was a 14-month-old boy who experienced prolonged bleeding after myringotomy. Endoscopic examination revealed redness and swelling in the left ear. Computed tomography (CT) showed a mastoid cavity filled with soft tissue and bone destruction. Seven days after the first consultation, the patient developed a fever and gait disturbances and was hospitalized. Enhanced CT showed bone destruction of the left ilium and fourth lumbar vertebra, and a tumor lesion around the left ilium. Magnetic resonance imaging demonstrated a tumor extending from the left petrous bone to the cerebellopontine angle. The patient underwent a mastoidectomy and tumor biopsy. Histopathological examination of the tumor revealed a myeloid sarcoma, suggestive of acute myeloid leukemia. The patient was subsequently diagnosed with AMKL. The patient underwent standard multi-agent chemotherapy, followed by allogeneic hematopoietic stem cell transplantation. At the 50-month follow-up, the patient continued to be in remission. In cases with unusual symptoms such as continuous bleeding after myringotomy, temporal bone CT scans should be promptly considered to identify tumors or hematological diseases.

## Introduction

Acute megakaryoblastic leukemia (AMKL) is a rare subtype of acute myeloid leukemia (AML) that was classified as M7 according to the French-American-British classification of AML in 1985 [1]. AMKL is observed in 7-10% of children with AML [2] and in only 1% of adults with AML [3]. Although the prognosis of Down syndrome-associated pediatric AMKL (DS-AMKL) is excellent [3], that of pediatric AMKL without Down syndrome (non-DS-AMKL) is heterogeneous [4].

Extramedullary involvement in AML, referred to as myeloid sarcoma or granulocytic sarcoma, represents the tumor-forming proliferation of leukemic blasts occurring outside the bone marrow and may precede or accompany systemic leukemia [5,6]. Extramedullary AMKL is extremely rare [5], and only a few cases have been reported in the temporal bone [5,6]. Such lesions may clinically mimic primary solid tumors or inflammatory diseases of the temporal bone, potentially leading to a delayed diagnosis.

Here, we present the clinical history, examinations, operations, and treatment details of a patient with AMKL who underwent mastoidectomy for biopsy and was treated with induction chemotherapy, intensive chemotherapy, and allogeneic hematopoietic stem cell transplantation.

## Case presentation

A 14-month-old boy visited the otolaryngology clinic because of frequent ear touching, which was observed over two weeks. Redness and swelling of the left tympanic membrane were observed, and the patient underwent myringotomy of the left eardrum at the clinic for suspected acute otitis media; however, bleeding from the left eardrum persisted. A surgical pad was placed for hemostasis; however, oozing persisted for several days. A tumor or vascular abnormality was suspected. Although hemostasis was difficult to achieve, oozing was ultimately controlled, and the patient was referred to our department.

**Figure 1:**
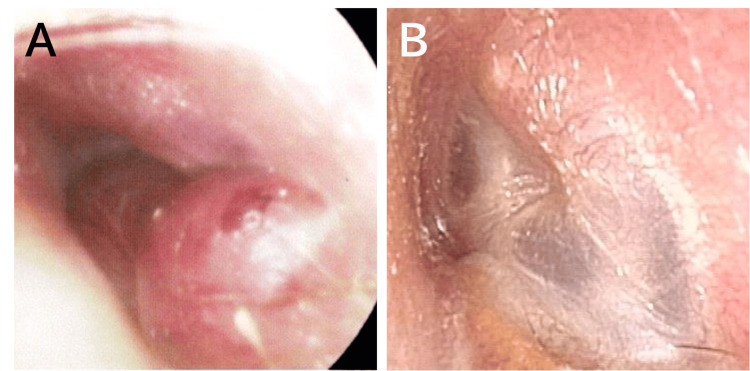
A pre- and post-treatment endoscopic examination of the left ear canal. (A) Pre-treatment. Redness and swelling observed in the eardrum; (B) Post-treatment. The redness and swelling were reduced.

The patient's medical history was unremarkable. No birth abnormalities or family history were noted. Endoscopy revealed redness and swelling of the left eardrum (Figure 1A). Conditioned-orientation audiometry (COR) via sound -field speakers ranged from 65 dB to 75 dB. The patient was crying during the assessment and appeared to have hearing loss.

**Figure 2:**
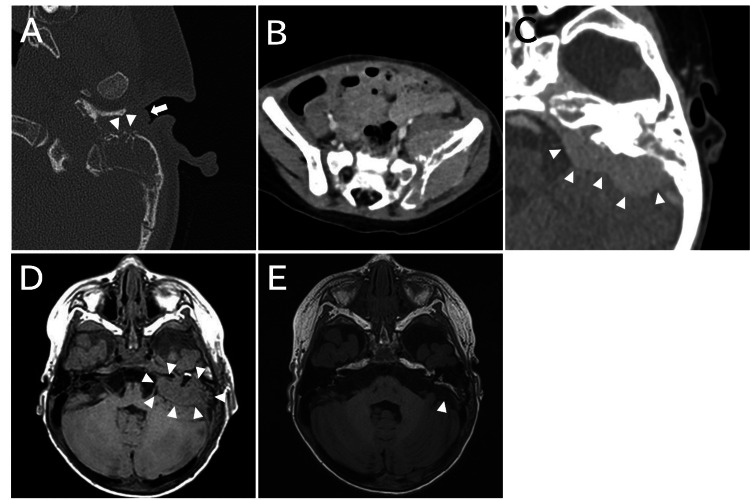
Pre- and post-treatment imaging. (A) Pretreatment axial CT scan of the temporal bone. The soft tissue fills the mastoid cavity, the posterior wall of the external auditory canal is destroyed (arrowheads), and the soft tissue extends into the external auditory canal (arrow); (B) Pretreatment axial enhanced CT scan of the left atrium. Bone destruction of the left ilium and a tumor lesion around the left ilium are observed (arrowheads); (C) Pretreatment axial enhanced CT of the head. A tumor lesion is observed in the posterior cranial fossa; (D) Pretreatment axial T1-weighted MRI of the head. A tumor lesion extending from the left petrous bone to the cerebellopontine angle is observed (arrowheads). Brain metastases are not observed. (E) Post-treatment axial T1-weighted MRI of the head shows tumor shrinkage (arrowhead).

Seven days after the first consultation, the patient developed a fever and gait disturbances and was hospitalized. Blood tests at this hospitalization showed a WBC count of 18410/µL (neutrophils 46.6%, lymphocytes 47.2%, no atypical lymphocytes), hemoglobin of 10.3 g/dL, platelet count of 229 × 103/µL, and C-reactive protein of 4.05 mg/dL. Computed tomography (CT) revealed a mastoid cavity filled with soft tissue and bone destruction of the mastoid with a periosteal reaction (Figure 2A). The posterior wall of the external auditory canal was destroyed, and the soft tissue extended into the external auditory canal. The bony wall of the posterior fossa was destroyed and extended into the posterior cranial fossa. No destruction of the bony labyrinth or ossicles was observed. An enhanced CT scan showed bone destruction of the left ilium and fourth lumbar vertebra, and a tumor lesion around the left ilium (Figure 2B). An enhanced CT and magnetic resonance imaging (MRI) revealed a tumor extending from the left petrous bone to the cerebellopontine angle and posterior cranial fossa (Figures 2C, 2D). The auditory and facial nerves were compressed ventrally. Brain metastases were not observed. Diffusion-weighted imaging showed iso-intensity within the brain parenchyma and no decrease in the apparent diffusion coefficient.

**Figure 3:**
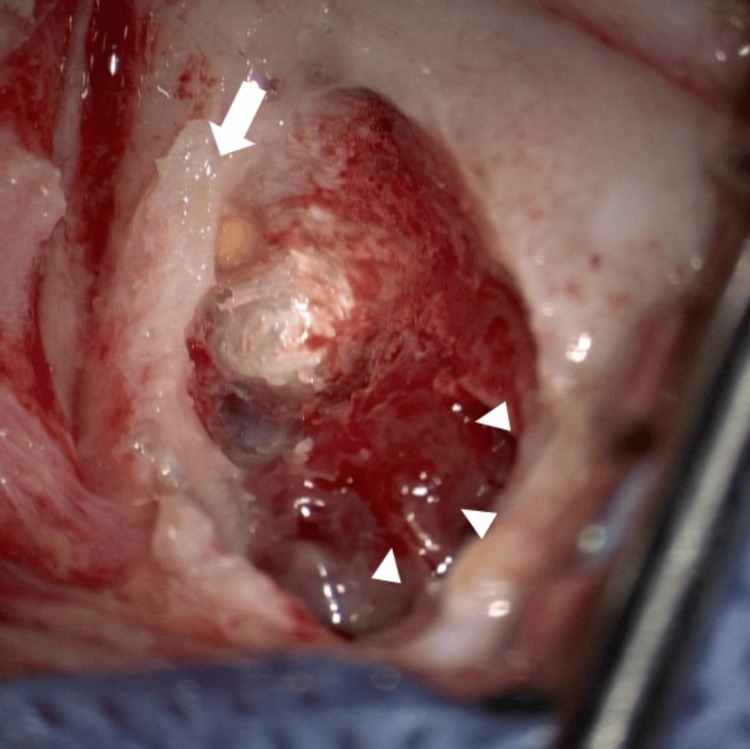
Intraoperative image after mastoidectomy. The red and hemorrhagic tumor was seen in the mastoid (arrowhead). Arrow: posterior wall of the external auditory canal.

**Figure 4:**
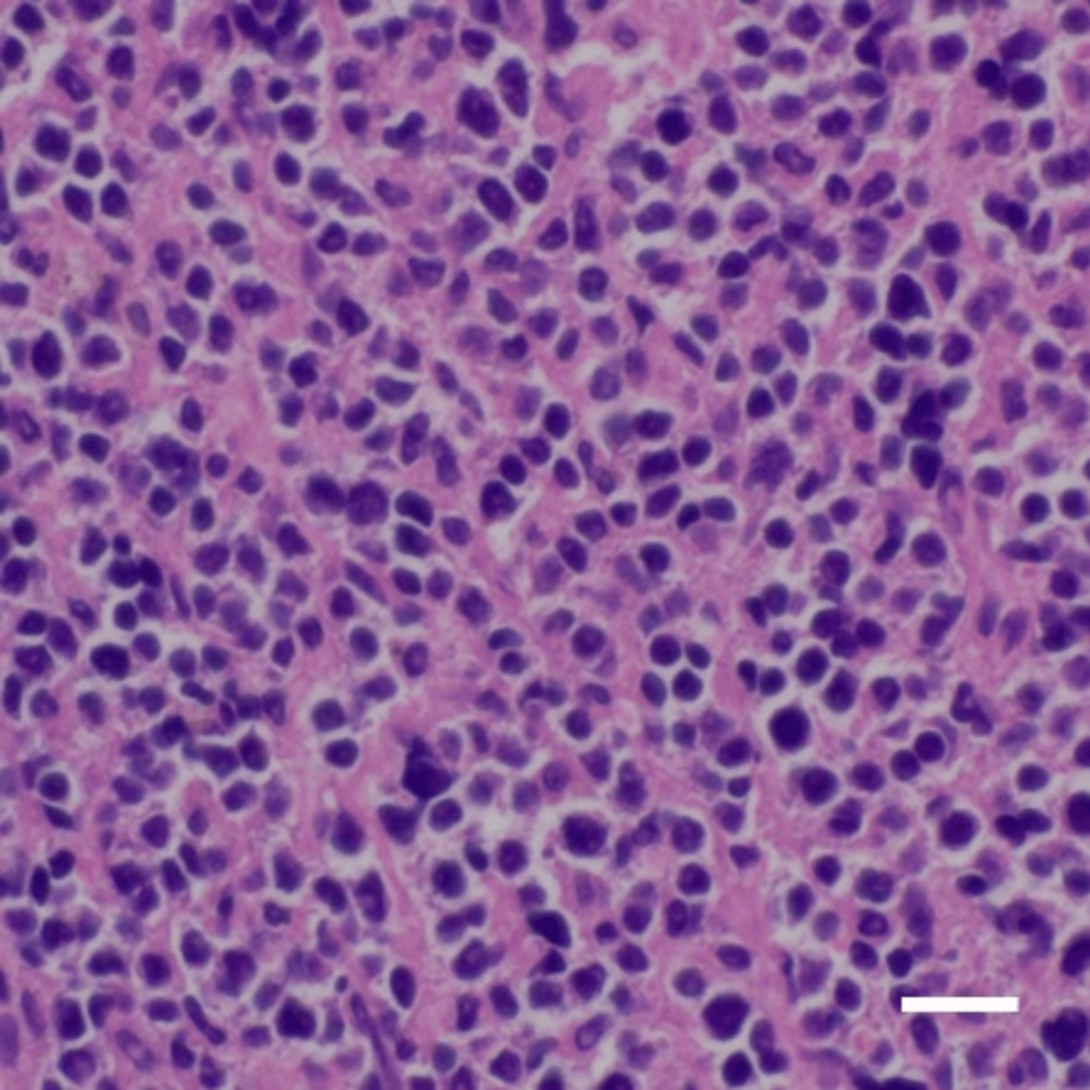
Hematoxylin and eosin staining of the specimen acquired from the temporal bone. Atypical cells with a small amount of cytoplasm proliferate in a solid pattern (bar: 20 µm).

The patient underwent mastoidectomy and tumor biopsy to establish a definitive diagnosis. A hemorrhagic mass lesion was found in the lower part of the mastoid cavity (Figure 3), which required additional time to control the bleeding. Rapid intraoperative pathological examination revealed that the lesion was malignant. Histopathological examination of the tumor revealed myeloid sarcoma- with CD33 and CD34 positivity, suggesting an extramedullary tumor lesion of acute myeloid leukemia (Figure 4). In the bone marrow, 86.4% blasts were detected and were negative for myeloperoxidase (MPO) staining. Flow cytometry of the bone marrow aspirate revealed that the tumor cells were immunopositive for CD33, CD117, CD41, CD61, and CD56. The patient was diagnosed with AMKL. Karyotype analysis using G-banding identified complex structural and numerical aberrations in the chromosomes.

After the first course of induction chemotherapy according to the Japanese Pediatric Leukemia/Lymphoma Study Group AML05 trial [7], complete hematological remission was achieved with a reduction in extramedullary lesions, including those in the mastoid cavity. However, after the second course of induction chemotherapy, a small number of leukemic cells remained in the bone marrow, as detected using in situ fluorescence hybridization. After four additional courses of consolidation therapy, including fludarabine, cytarabine, and granulocyte colony-stimulating factor in combination with gemtuzumab ozogamicin [8], the bone marrow leukemic cells became undetectable, and the patient underwent allogeneic bone marrow transplantation from an human leukocyte antigen (HLA)-matched unrelated donor using myeloablative conditioning using busulfan and melphalan.

Following treatment, hearing in the affected ear improved to the same level as that in the contralateral ear, except at high frequencies. Redness and swelling of the left eardrum (Figure 1B) were reduced, and the tumor shrank (Figure 2E). The patient has remained in complete remission for 50 months after transplantation.

## Discussion

Pediatric patients with AMKL are generally classified into two major subgroups: DS-AMKL and non-DS-AMKL [6,7]. DS-AMKL has been reported to respond better to chemotherapy than non-DS-AMKL, which has also demonstrated poor outcomes [2,7,8]. Our patient was diagnosed with non-DS-AMKL and exhibited extramedullary lesions in the temporal bone and left ilium. Extramedullary lesions of AML are known as myeloid sarcomas and are rare in AMKL [9]. Extramedullary AMKL exhibits several characteristics [5]: it is associated with younger age, particularly in individuals aged < 2 years; it is more common in females, whereas AML is more common in males; and it is more likely to involve the bone. One possible explanation is that megakaryoblasts may have a higher affinity for bone under the influence of megakaryocyte-associated cytokines [5].

Although AMKL is characterized by > 20% blasts in the peripheral blood or bone marrow [8], some patients with AMKL may present with < 20% blasts [10]. In addition, immunostaining for MPO and CD41 in the bone marrow is useful for diagnosing AMKL, with MPO staining typically negative and CD41 staining positive [8],. In this patient, blasts were not detected in the peripheral blood; however, 86.4% of the blasts were detected in the bone marrow, confirming the diagnosis. MPO and CD41 staining of the bone marrow were negative and positive, respectively.

Although myeloid sarcomas are rare, an early and accurate diagnosis is necessary to improve functional outcomes. Reports indicate that myeloid sarcomas are often misdiagnosed as lymphomas [11]. When abnormal findings are observed in the external auditory canal and eardrum, unlike in acute otitis media or otitis media with effusion, imaging techniques, such as CT, should be considered for early diagnosis.

Destructive temporal bone diseases in infancy include cholesteatoma and malignancies such as rhabdomyosarcoma, Langerhans cell histiocytosis, and Ewing sarcoma. In the current case, a red eardrum and persistent oozing were observed after myringotomy, which are uncommon in cholesteatoma. Therefore, a malignant tumor was suspected. Histopathological examination was required for a definitive diagnosis. The patient was suspected of having a tumor or vascular abnormality of the temporal bone, and an early CT scan was performed.

Considering the risk of difficult hemostasis, the risk of patient movement during the procedure, especially in pediatric patients, and the need to harvest sufficient specimens for a definitive diagnosis, we opted for a biopsy under general anesthesia rather than as an outpatient procedure. When a malignant temporal bone tumor is suspected in pediatric patients, a biopsy under general anesthesia should be performed as soon as possible to avoid the risk of bleeding and to obtain adequate lesion sampling. When a tumor is hemorrhagic in children, controlling the bleeding may be difficult without sedation.

## Conclusions

This was a case of pediatric AMKL with myeloid sarcoma of the temporal bone. When unusual symptoms, such as continuous bleeding after myringotomy, are observed, temporal bone CT scans should be promptly considered to identify tumor lesions or blood diseases. Mastoidectomy and biopsy under general anesthesia are recommended for a definitive diagnosis when a malignant temporal bone tumor is suspected in pediatric patients.
